# The Challenging Management of Gallbladder Masses: A Case of Xanthogranulomatous Cholecystitis and Diffuse Biliary Hamartomatosis Mimicking Gallbladder Metastatic Cancer

**DOI:** 10.1155/cris/1343763

**Published:** 2026-06-30

**Authors:** Chiara D’Alterio, Stefania Brozzetti

**Affiliations:** ^1^ Department of Surgery, Umberto I University Hospital, Sapienza University of Rome, Viale del Policlinico 155, Rome, 00161, Italy, uniroma1.it

**Keywords:** case report, gallbladder cancer, multicystic liver disease, multiple biliary hamartomas, xanthogranulomatous cholecystitis

## Abstract

**Background:**

Xanthogranulomatous cholecystitis (XGC) is a rare form of chronic cholecystitis characterized by progressive fibrosis of the gallbladder wall. Its clinical, laboratory, and radiological features often mimic gallbladder carcinoma (GC), making preoperative diagnosis challenging. A definitive diagnosis is usually established by histopathological examination. As XGC may occasionally coexist with malignancy, surgical resection is generally recommended.

**Case Presentation:**

A 66‐year‐old woman presented with weight loss, epigastric discomfort, and a palpable mass in the right upper quadrant. Imaging revealed diffuse gallbladder wall thickening and a 33‐mm vascularized mass in the gallbladder fundus, with suspected extension to the liver, transverse colon, and abdominal wall, strongly suggesting GC. Multiple hepatic cysts were also identified. Following multidisciplinary evaluation, laparoscopic exploration was performed. Hepatic and colonic infiltration, distortion of Calot’s triangle, regional lymphadenopathy, and diffuse millimetric liver lesions initially suggested GC with intrahepatic metastases. However, histological examination of biopsied lesions excluded malignancy. After a 4‐month recovery from COVID‐19, the patient experienced clinical improvement and weight gain. Follow‐up computed tomography (CT) showed regression of both the gallbladder mass and hepatic involvement, supporting a benign inflammatory process. During subsequent laparotomy, frozen‐section analysis of the colonic infiltration confirmed inflammatory tissue. Radical cholecystectomy with en bloc resection of the adjacent liver parenchyma was performed. Final histopathology revealed diffuse XGC associated with diffuse biliary hamartomatosis.

**Conclusion:**

This case highlights the diagnostic challenge of distinguishing XGC from GC, particularly in the presence of biliary hamartomatosis. A stepwise diagnostic approach and multidisciplinary decision‐making were essential to ensure accurate diagnosis and appropriate management.

## 1. Background

Xanthogranulomatous cholecystitis (XGC) is an uncommon benign gallbladder disease resulting from severe chronic inflammation. It was first described as a distinct pathological entity in 1981 by Goodman and Ishak [1]. XGC accounts for ~0.7%–13.2% of all cases of cholecystitis [2], with a reported prevalence ranging from 1% to 9% worldwide [3]. The condition predominantly affects older adults, typically during the sixth and seventh decades of life, although its gender distribution remains controversial [4]. Histologically, XGC is characterized by the rupture of the Rokitansky–Aschoff sinuses, resulting in the extravasation of bile and cholesterol into the gallbladder wall. This process triggers a xanthogranulomatous inflammatory reaction characterized by the accumulation of lipid‐laden macrophages, multinucleated giant cells, and progressive fibrosis. The inflammatory process may extend beyond the gallbladder, involving adjacent structures such as the liver and porta hepatis [1]. The clinical, laboratory, and radiological features of XGC may closely resemble those of gallbladder carcinoma (GC), making preoperative diagnosis particularly challenging. Furthermore, inflammatory changes may occasionally be associated with elevated serum tumor markers, including carcinoembryonic antigen (CEA), carbohydrate antigen 19‐9 (CA19‐9), and alpha‐fetoprotein (AFP), further contributing to diagnostic uncertainty. Although XGC is a benign condition, concomitant GC has been reported in a small proportion of cases [5, 6]. Consequently, differentiating XGC from GC and determining the most appropriate management strategy remain significant clinical challenges. We report the case of a 66‐year‐old woman with XGC associated with biliary hamartomatosis, initially suspected to have metastatic GC. This case report was prepared in accordance with the SCARE (Surgical CAse REport) criteria [7]. Written informed consent was obtained from the patient for the publication of this report and the accompanying images. We also review the current literature on the differential diagnosis and management of XGC and GC and discuss the clinical implications of this unusual presentation.

## 2. Case Presentation

The patient was a 66‐year‐old Caucasian woman with a history of scleroderma, an autoimmune fibrosing disorder treated with corticosteroids, systemic hypertension, and severe chronic obstructive pulmonary disease related to long‐term smoking (~20 cigarettes per day for 40 years). Her medical history also included anxiety–depressive disorders and claustrophobia. Previous abdominal surgery consisted of an open appendectomy and a cesarean section. Family history was negative for significant chronic or neoplastic diseases. In February 2022, she presented to the emergency department with upper abdominal pain and nausea, but without vomiting or fever. Laboratory tests were unremarkable. Abdominal ultrasonography revealed multiple hepatic cysts, the largest measuring 16 and 10 mm in segments II and IV, respectively, mild dilation of the common bile duct (8 mm), and gallbladder wall thickening with microlithiasis and irregular vascularized thickening of the fundus (8 mm), raising suspicion for malignancy. Laparoscopic cholecystectomy was recommended, but the patient declined surgery. In June 2022, contrast‐enhanced abdominal computed tomography (CT), performed at another institution, demonstrated an enlargement of the gallbladder fundus lesion to 20 mm, with apparent hepatic infiltration, perivisceral inflammatory changes, and a 50‐mm septated fluid‐filled lesion in segment V. Two endoscopic ultrasound‐guided transduodenal biopsies were negative for malignancy. The patient was referred to our institution in October 2022 because of persistent abdominal discomfort and an unintentional weight loss of 8 kg (BMI 19.7 kg/m^2^). Physical examination revealed a palpable mass in the right upper quadrant, with a negative Murphy’s sign. Contrast‐enhanced CT demonstrated a 33‐mm exophytic mass arising from the gallbladder fundus, infiltrating the adjacent liver parenchyma, anterior parietal peritoneum, and transverse colon, with associated perivisceral fat stranding extending to the hepatic flexure. The lesion showed marked contrast enhancement. The gallbladder wall appeared thickened and bilaminar, without pericholecystic fluid. Perihilar lymphadenopathy and a slight reduction in segment V fluid collection were also observed (Figure 1). Magnetic resonance imaging (MRI) was not feasible because of severe claustrophobia. Laboratory tests and tumor markers were within normal limits (CA19‐9 12 U/mL, AFP 3.2 ng/mL; CEA 1.8 ng/mL). Given the high suspicion of locally advanced GC (cT4), the multidisciplinary hepatobiliary tumor board recommended diagnostic laparoscopy with the intent to proceed to radical resection and lymphadenectomy, in accordance with the ESMO guidelines for biliary tract cancers [8]. Laparoscopy revealed dense adhesions involving the omentum, colon, diaphragm, and inferior liver surface, completely obscuring the gallbladder and Morrison’s pouch. Segment V was distorted by a whitish fungating lesion, while the liver surface was scattered with multiple miliary subcentimetric white nodules (Figure 2). These findings raised concern for diffuse intrahepatic metastases; therefore, the procedure was discontinued, and multiple biopsies were obtained from clustered lesions along the anterior hepatic margin for histopathological and immunohistochemical evaluation. Unexpectedly, definitive pathology demonstrated biliary cysts. A second laparoscopic procedure was planned to biopsy the gallbladder mass; however, the patient subsequently developed COVID‐19 infection and experienced a prolonged recovery because of her underlying pulmonary disease, resulting in surgical delay. In March 2023, a clinical reassessment showed weight gain and overall improvement in her general condition. Repeat contrast‐enhanced CT demonstrated a reduction in the size of the gallbladder fundus lesion (26 mm), with decreased vascularization and peritoneal involvement, together with regression of the segment V lesion (20 mm), findings suggestive of a benign inflammatory process. Consequently, the operative strategy was revised to open exploration with intraoperative frozen‐section analysis and liver ultrasonography to guide the extent of resection. Through a right subcostal incision extended across the midline, adhesiolysis was carefully performed. Frozen‐section analysis of an incisional biopsy from the infiltrated transverse colon demonstrated granulomatous inflammatory tissue without evidence of malignancy. Following colonic repair, a radical cholecystectomy with an en bloc resection of the gallbladder bed was carried out (Figure 3). Liver transection was performed using an ultrasonic dissector, and the resection surface was secured with sutures, clips, and a hemostatic matrix to achieve hemostasis and biliostasis. The postoperative course was complicated by a bile leak on postoperative day 8. Endoscopic cholangiography demonstrated an intact biliary tree and identified a leak originating from the hepatic resection surface. Endoscopic sphincterotomy was performed, resulting in a progressive reduction of the drain output. The patient was discharged in good clinical condition with the drain in place and was followed in the outpatient setting until its removal on postoperative day 21. The final histopathological examination revealed XGC with inflammatory adhesions involving the adjacent liver parenchyma and focal abscess formation at the liver–gallbladder interface, associated with multiple biliary hamartomas. At the 18‐month clinical and ultrasonographic follow‐up, the patient remained asymptomatic and in good overall condition.

**Figure 1:**
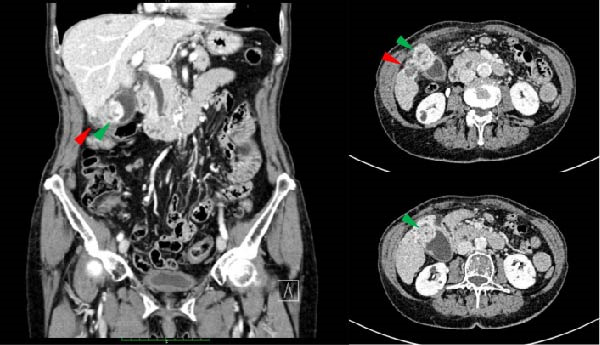
Contrast‐enhanced computed tomography scans (Oct2022). The green arrows indicate the exophytic mass of the gallbladder fundus (33 mm of diameter) infiltrating the liver parenchyma, the anterior parietal peritoneum, and the transverse colon. The gallbladder wall was thickened and bilaminar, without pericholecystic fluid. The red arrows indicate the fluid lesion localized in segment V. The blue arrow indicate one of the liver cysts.

**Figure 2:**
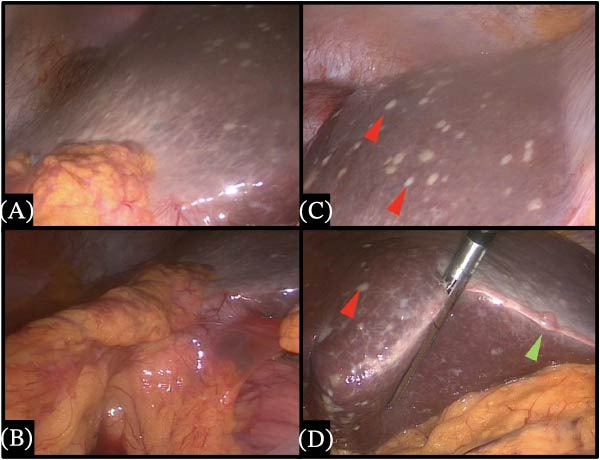
(A, B) Extensive adhesions involving the omentum and colon along with the diaphragmatic and inferior surfaces of the liver, completely obscuring the gallbladder and the Morrison’s space. (C, D) Diffuse parenchyma involvement by multiple miliary subcentimetric white lesions. The red arrows indicate some of the intrahepatic miliary lesions. The green arrow indicates the cluster of lesions that has been resected for pathological examination.

**Figure 3:**
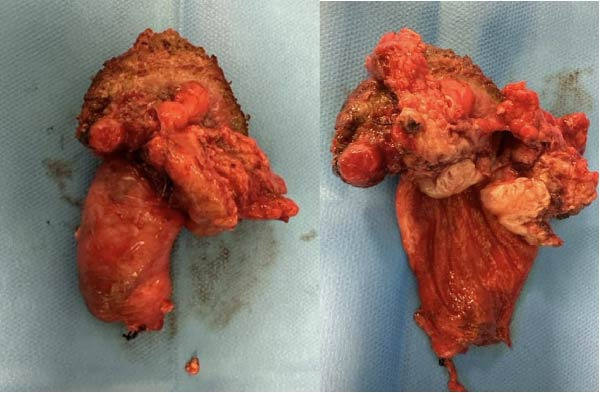
The surgical specimen: cholecystectomy with en bloc resection of the gallbladder bed.

## 3. Discussion

XGC remains a poorly understood and diagnostically challenging condition. Patients may remain asymptomatic for prolonged periods or present with nonspecific symptoms, including nausea, vomiting, fever, jaundice, anorexia, right upper quadrant pain or tenderness, a palpable mass, or a positive Murphy’s sign. Although benign, XGC may exhibit locally aggressive inflammatory behavior, leading to complications such as perforation, abscess formation, and fistulae, and frequently extends to adjacent organs [9]. The exact etiology of XGC remains unclear, but the condition is thought to result from bile extravasation into the gallbladder wall in the setting of gallstones, biliary obstruction, or cholestasis. The inflammatory process typically originates in the Rokitansky–Aschoff sinuses and progresses to a granulomatous reaction that may lead to the formation of submucosal abscesses [1]. Subsequently, marked proliferative fibrosis develops within the gallbladder wall, often accompanied by asymmetric thickening and multiple yellowish nodules [1]. Serosal perforation and extension of the inflammatory process may result in dense adhesions involving surrounding structures, including the liver, duodenum, and transverse colon [9]. In our patient, extensive XGC obscured the gallbladder–liver interface and infiltrated adjacent tissues and organs, making preoperative differentiation from malignancy extremely difficult. Because XGC can closely mimic GC, accurate diagnosis is essential for appropriate management. In 2021, an International Expert Consensus proposed the Gallbladder Reporting and Data System (GB‐RADS), a risk stratification tool for gallbladder wall thickening detected by ultrasonography in nonacute settings, aimed at identifying lesions suspicious for GC and guiding further imaging investigations [10]. However, XGC is frequently classified as high‐risk according to GB‐RADS criteria [10]. Several studies have investigated clinical, biochemical, and radiological features that may help distinguish XGC from GC [9, 11–15]. Hale et al. [16] reported that both conditions may present with a “halo sign” (a hypodense curvilinear band), intrahepatic bile duct dilatation, and loss of the gallbladder–liver interface. In contrast, preservation of a continuous mucosal line and the absence of pericholecystic fluid are more suggestive of XGC [16]. MRI with magnetic resonance cholangiopancreatography (MRI/MRCP) provides the highest diagnostic accuracy [17], although MRI could not be performed in our patient because of severe claustrophobia. To date, no clinical, laboratory, or imaging finding has demonstrated sufficient accuracy to reliably establish the diagnosis of XGC preoperatively; therefore, a definitive diagnosis still relies on the histopathological examination. Importantly, a metaplasia–dysplasia–carcinoma sequence has been described in XGC [18, 19], and concomitant GC may occasionally occur. The reported incidence of coexisting GC ranges from 0% to 20%, while misdiagnosis may result in inappropriate over‐ or undertreatment in ~10.2% of cases [16]. Laparoscopic cholecystectomy remains the standard treatment for benign gallbladder disease, including XGC; however, severe inflammation, dense adhesions, and fistula formation require conversion to open surgery in ~26% of patients. Conversely, the standard treatment for locally advanced GC includes radical cholecystectomy with en bloc resection of liver segments IVb and V, regional lymphadenectomy, resection of the extrahepatic bile duct when indicated, and removal of any involved adjacent organs [16]. For this reason, preoperative tissue sampling by endoscopic ultrasound‐guided biopsy or intraoperative frozen‐section analysis is recommended before undertaking extensive surgical resection, as outlined in the management algorithm proposed by Hale et al. [16] for patients with sonographically suspicious XGC or GC. In our patient, the presence of diffuse biliary hamartomatosis further complicated the differential diagnosis. To the best of our knowledge, no previous reports have described an association between diffuse biliary hamartomatosis and XGC. Biliary hamartomas are uncommon benign malformations of the intrahepatic bile ducts [20], first described by Von Meyenburg [21] and commonly referred to as Von Meyenburg complexes. These lesions belong to the spectrum of fibropolycystic liver diseases and arise from abnormal embryological development of the ductal plate, the cylindrical layer of cells surrounding the portal vein branches that subsequently differentiates into the intrahepatic bile ducts [20]. Their prevalence has been estimated at ~5.6% in adults [22]. Clinically, biliary hamartomas are usually asymptomatic and are most often detected incidentally during imaging studies, surgical procedures, or autopsy [20]. Histologically, they consist of irregular and disorganized small bile ducts embedded within a fibrocollagenous stroma, showing variable degrees of hyalinization. The ducts may exhibit different degrees of dilatation, contain inspissated bile, and typically do not communicate with the normal biliary tree. In cases of marked ductal dilatation, adjacent ducts may coalesce to form cystic spaces containing residual connective tissue, which can appear as endocystic polypoid projections [23]. Macroscopically, biliary hamartomas appear as multiple small white–gray nodules, usually located in a subcapsular distribution or scattered throughout the hepatic parenchyma. Most lesions measure less than 5 mm in diameter, although they may occasionally coalesce into larger cystic structures [23]. Owing to their appearance and distribution, these lesions may easily be mistaken for hepatic metastases [24–27]. MRI can assist in differentiating biliary hamartomas from metastatic lesions. On heavily T2‐weighted sequences, hamartomas typically demonstrate persistently high signal intensity and remain markedly hyperintense compared with metastases [23]. Because MRI provides superior soft‐tissue contrast resolution compared with CT, it is more sensitive in detecting small T2‐hyperintense nodules. When numerous lesions are present, the liver may exhibit the characteristic “starry sky” appearance of diffuse biliary hamartomatosis [23]. Unfortunately, an MRI could not be performed on our patient because of severe claustrophobia, limiting the preoperative diagnostic work‐up. The strong preoperative and intraoperative suspicion of malignancy initially discouraged intraoperative frozen‐section examination during the first laparoscopic exploration. However, subsequent clinical and radiological reassessments suggested a benign inflammatory process, which was ultimately confirmed by frozen‐section analysis during the definitive laparotomy. Resection of the gallbladder bed was therefore performed because of inflammatory hepatic infiltration rather than oncological indications, and lymphadenectomy was deemed unnecessary. This case highlights several pitfalls in the evaluation and management of gallbladder masses, including the deceptively infiltrative appearance of XGC, the challenging differential diagnosis between diffuse biliary hamartomatosis and hepatic metastases, and the important role of MRI in achieving an accurate preoperative assessment. In this complex clinical scenario, a careful stepwise diagnostic approach and multidisciplinary decision‐making were essential to ensure an accurate diagnosis and appropriate management.

## 4. Conclusion

XGC should always be considered in the differential diagnosis of gallbladder masses suspicious for malignancy. Although it is a benign condition, timely recognition and appropriate treatment are essential because of its potential for severe complications and its occasional association with GC. This case highlights the importance of a multidisciplinary approach involving surgeons, radiologists, and pathologists in both preoperative assessment and intraoperative decision‐making. In particular, intraoperative frozen‐section analysis and a staged surgical strategy, tailored according to histopathological findings, represent valuable tools for avoiding unnecessary extensive resections while ensuring appropriate management.

## Author Contributions

Chiara D’Alterio and Stefania Brozzetti conceived and designed the study. Chiara D’Alterio and Stefania Brozzetti collected the clinical data. Stefania Brozzetti performed the surgical procedures. Chiara D’Alterio drafted the manuscript, and Stefania Brozzetti critically revised it for important intellectual content.

## Funding

Open access publishing facilitated by Universita degli Studi di Roma La Sapienza, as part of the Wiley ‐ CRUI‐CARE agreement.

## Disclosure

All scientific content, interpretations, and conclusions were conceived, reviewed, and approved by the authors, who take full responsibility for the final version of the manuscript.

## Consent

Written informed consent was obtained from the patient for the publication of this case report and the accompanying images.

## Conflicts of Interest

The authors declare no conflicts of interest.

## Data Availability

Data sharing is not applicable to this article, as no new data were created or analyzed in this study.
